# Large-Area Mapping of Voids and Dislocations in Basal-Faceted Sapphire Ribbons by Synchrotron Radiation Imaging

**DOI:** 10.3390/ma16196589

**Published:** 2023-10-07

**Authors:** Tatiana S. Argunova, Victor G. Kohn, Jae-Hong Lim, Vladimir M. Krymov, Mikhail Yu. Gutkin

**Affiliations:** 1 Ioffe Institute, Russian Academy of Sciences, Polytekhnicheskaya St. 26, 194021 St. Petersburg, Russia; v.krymov@mail.ioffe.ru; 2 National Research Centre ‘Kurchatov Institute’, Kurchatov Sqr., 1, 123182 Moscow, Russia; kohnvict@yandex.ru; 3 Pohang Accelerator Laboratory, Pohang, Gyeongbuk 37673, Republic of Korea; limjh@postech.ac.kr; 4 Institute for Problems in Mechanical Engineering, Russian Academy of Sciences, 199178 St. Petersburg, Russia; m.y.gutkin@gmail.com; 5 Institute of Advanced Data Transfer Systems, ITMO University, 197101 St. Petersburg, Russia

**Keywords:** sapphire ribbons, gas voids, dislocations, synchrotron radiation imaging

## Abstract

The understanding of structural defects in basal-faceted sapphire ribbons was improved through X-ray imaging at a synchrotron source. The combination of phase contrast and X-ray diffraction makes it possible to visualize and characterize both gas voids and dislocations in the bulk of the ribbons grown by the Stepanov–LaBelle technology. Dislocations were directly related to gas voids. X-ray diffraction topography was employed to investigate the distribution, configurations, and character of the dislocations. The formation of voids of irregular shapes was detected by large-area mapping with spatial resolution in the μm range. Computer simulations of the experimental phase contrast images of microvoids were performed. The sizes of the spherical microvoids were determined. The results are discussed with reference to the available data on the emission of dislocations from the voids. The evolution of the shape, size, and arrangement of the voids during growth provides clues on the formation of block structure in basal-faceted sapphire ribbons.

## 1. Introduction

X-ray topography (XRT) and phase contrast imaging (PCI) methods are optimally combined with each other at the third-generation synchrotron radiation (SR) sources. Natural narrow angular collimation of SR is essential for high angular resolution of X-ray topographs. At the same time, the high spatial coherence makes it possible to observe (through the interference phenomenon) an additional phase shift variable over the cross-section of the beam that has passed through an inhomogeneous object [1,2].

PCI in combination with XRT exploits instrumental setups on special imaging beamlines, which allow the registration of both types of images using monochromatic or white SR beams. In addition, various X-ray detectors are available for recording such images. Unique facilities provide real-time experiments that shed some light on the effects of growth, solidification, or melting of crystalline materials [3,4,5].

Moreover, XRT and PCI can be applied sequentially with strict reference to the same area of a specimen. In this case, the spectrum and detectors change depending on the purpose of the experiment. For Bragg adjustment using the white SR beam, so that diffraction conditions are satisfied for a set of Bragg planes, a flat detector is the ideal choice. A large-area panel made with CMOS technology registers several reflections from different crystal planes at once. However, the best results are obtained with high-resolution X-ray films. As for the intensity variation caused by a variable phase shift, it is recorded using a charge-coupled device (CCD) with a micrometer pixel size. First, the X-ray intensity is converted into visible light from a crystal scintillator. Then the lens magnifies the transmitted light onto the detector. A correspondence between crystal lattice defects and inhomogeneities (pores, inclusions, and micro-cracks) is established by comparing topographs with phase contrast images [6,7,8,9,10].

Real crystals are not uniform across their area. To make good products, it is important to establish not only the types but also the distribution of defects. Only the PCI technique [1,2] with a fairly large field of view is suitable for this purpose. The comparison of PCI with other methods such as coherent diffraction imaging, ptychography, and microscopy shows its effectiveness for analyzing large sample areas. The experimenter can map defects and compare the XRT and PCI maps with account for scale disparities and differences in the nature of the contrasts. We emphasize that phase contrast images are interference patterns. The real size of a micro-object can be obtained only by determining the change in the phase. At the same time, the interpretation of a topograph is often possible without the assistance of the X-ray diffraction theory.

The present paper describes the investigation of gas voids and dislocations in sapphire crystals grown by the edge-defined film-fed growth (EFG) method invented by H. LaBelle and even earlier by A.V. Stepanov [11,12]. Our goal was to highlight the contribution that XRT and PCI can make (through the use of SR) for better understanding of defect formation in basal-faceted sapphire ribbons. The high demand for shaped sapphire products (see, e.g., [13]) encourages researchers to devote more attention to their study. Nevertheless, some problems arising in the growth of certain types of articles remain poorly understood. In particular, for the mass production of substrates, EFG does not look as efficient as other technologies. The main disadvantages of basal-faceted ribbons are gas voids and higher dislocation densities compared with Czochralski and Kyropoulos grown crystals [14,15]. In the meantime, a ribbon has a smooth surface and regular shape. It can be used for a substrate in its as-grown state.

In earlier works, it was observed that gas voids played an important role in the generation of dislocations in sapphire ribbons [16,17]. A model for the block structure generation in basal-faceted ribbons was proposed and confirmed by calculations of thermal stress in a growing crystal [16]. In the present paper, special attention is paid to the characteristics of gas voids and dislocations. An unambiguous relationship between them is revealed. Some specific features of the voids associated with dislocation emission from a void are discussed. As will be shown, the combination of applied imaging techniques is a particularly suitable tool for this study.

## 2. Materials and Methods

The growth of basal-faceted sapphire ribbons by the Stepanov/EFG method took place in a thermal zone where heat shields were used. The growth rate was 1 mm/min. Laser adjustment of the seed relative to the pulling device and the shaper allowed the achievement of a high orientation accuracy. The orientation of ribbon surfaces deviated only slightly (1.0 ′–3.0′) from the (0001) plane. Optical microscopy showed no blocks. The as-grown surfaces were smooth and mirror-like. Specimens were cut perpendicular to the growth direction [$10\bar{1}0$] so that they contained the neck portion of a ribbon.

The imaging experiments were carried out at the Pohang Light Source (PLS) in Pohang, South Korea. The PLS operated with an electron energy of 3 GeV. The 6C beamline, devoted to imaging, featured a wiggler with the total generated power of 10^7^–10^9^ ph/s/mm^2^ at 150 mA run. The angular size of the source (as seen from a point in the specimen) was in the ~10 μ radian range, leading to the transverse coherence length of several tens of microns. The beam was monochromated by a multilayer mirror with an energy resolution of Δ*E*/*E* ≈ 2%. The topography and PCI experiments were carried out at photon energies of 15 and 23 keV, respectively. 

A Zyla CCD (Andor, Oxford Instruments, UK) was utilized to record the phase contrast images. The CCD had 2560 × 2160 pixel resolution and 6.5 × 6.5 μm^2^ pixel size. Therefore, the maximum sample area (the field of view, FOV) that the camera could image was 16.6 × 14.0 mm^2^. The image recording was preceded by a conversion step in which the LuAG:Ce scintillator converted the radiation into the light. An optical lens served as the coupling element between the scintillator and the CCD. When a light image was magnified by a factor of 20×, the pixel-to-object size ratio decreased. The effective pixel size and the FOV were reduced down to 0.32 μm and 819 × 691 μm^2^, respectively.

High-speed topographs were recorded on the large FOV (64 × 42 mm^2^) detector VHR CCD (Photonic Science, Saint Leonards, UK) with a pixel size of 16 μm. High-resolution images were taken on the fine-grain film Kodak M100. The typical exposure times were short due to the high intensity available at the 6C wiggler beamline. They ranged from several tens of seconds (on the film) to several milliseconds (on the VHR CCD). The comparison of topographs and phase contrast images could not be performed within the same experimental session due to differences in the setups and viewing angles. To achieve a better match, the phase contrast images were recorded when the sample was set in the azimuth position for X-ray diffraction.

## 3. Results

### 3.1. As-Grown Dislocations

The fact that single crystals of sapphire can be grown in the shape of basal-faceted ribbons raises questions as to the configuration of dislocations in such crystals. The study of this problem is a continuation of extensive experiments carried out by chemical etching, X-ray topography, and transmission electron microscopy methods. Initially, from a theoretical study of the crystal structure, possible slip systems in sapphire were proposed such as basal, prismatic, and pyramidal. For over 70 years of research, the existence of these slip systems has been clearly confirmed. From a topographic study, basal dislocations with 〈$11\bar{2}0$〉 Burgers vectors were revealed [18,19,20]. The existence of the prismatic slip system 〈$10\bar{1}0$〉, {$1\bar{2}10$} was well evidenced [21,22]. The reactions of dislocations were studied in detail (see, e.g., [20,22]).

Dislocation density in sapphire for application as a substrate does not exceed 10^3^ cm^−2^, which makes it possible to directly observe dislocations in single crystals grown by the heat exchange method [23] and the Czochralski [24], Verneuil [25], and Kyropoulos [26] processes. At the same time, relatively little is known about defects in profiled articles, especially ribbons. The problem of growing basal-faceted ribbons with low dislocation density has not been solved so far.

Thin ribbons with a wide basal face are characterized by high dislocation density and block structure [17]. Dislocations formed by thermal stresses set up in the solidified material during the cooling. Using a thermal field model, the authors of [16] calculated thermoelastic stresses arising in basal and prismatic slip planes. The authors arrived at the conclusion that in a thin basal-faceted ribbon, the stress acting in the basal slip system was very low (~0.1 MPa). A further conclusion was that blocks were formed from dislocations belonging to the prismatic slip system. This result has not yet been confirmed by experimental evidence. Since the yield stress *τ* decreases with increasing temperature and eventually becomes $\tau_{0001}<\tau_{1\bar{1}00}$ by a factor of 3–4 at *T* = 1000 °C [27], it is likely that basal slip is involved in stress reduction.

Here we present our results on XRT experiments with a basal-faceted ribbon of 31 × 1 × 265 (*W* × *H* × *L*) mm^3^ in size. The optimum thickness for a projection topograph depends upon the absorption of the material for the radiation used, namely, *μ t* = 1, where *μ* is the absorption coefficient and *t* is the specimen thickness. In our case, *λ* = 0.827 Å so that for sapphire, *μ* = 18.15 cm^−1^; therefore, the optimum thickness is 0.5 mm. The sample thickness was two times larger than the optimal value. However, *μ t* = 1.8 does not yet fall within the interval *μ t* = 2–5, where the dislocations can reveal both normal and reversed contrast [28].

**Figure 1:**
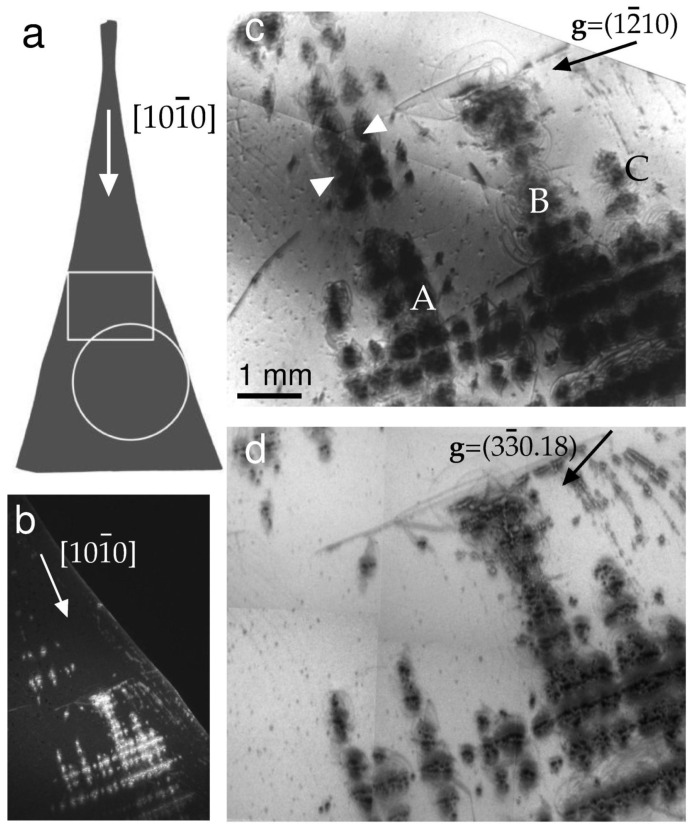
(**a**) Sketch of a sapphire specimen. The circle and the box show the areas imaged by XRT and PCI techniques, respectively. The arrow is the growth direction. (**b**) A high-speed topograph in back reflection taken with SR radiation (*λ* = 0.827 Å) on imaging detector VHR CCD, **g** = $3\bar{3}018$. (**c**) The topograph in transmission taken with Kodak M100 film. **g** = $1\bar{2}10$. (**d**) **g** = $3\bar{3}018$; Kodak M100.

The front view of the sample is schematically represented in Figure 1a. The arrow located in the neck portion is parallel to the direction of growth [$10\bar{1}0$]. In the sketch, the circle and box refer to the places imaged by SR XRT and PCI, respectively. Figure 1b shows a high-speed image taken on a VHR CCD detector. Features of the defect density and distribution were quickly visualized within ~2 × 10^−3^ s. The following characteristics were visible. First, the rows of dislocation pileups were parallel and perpendicular to the growth direction of the ribbon. Second, the pileups consisted of tangles associated with some dislocation sources. The number of the sources eventually increased with increasing distance from the seed. 

Figure 1c,d show topographs taken with Kodak M100 fine-grain film. In high-resolution images, dense tangles were formed by curved lines that were reminiscent of dislocation semiloops. To find basal dislocations in the ribbon, a representative configuration should be revealed by a topograph taken in any {$1\bar{2}10$} diffraction plane. Such a topograph of a ($1\bar{2}10$) reflection is shown in Figure 1c. The contrast arising from pileups labeled A, B, and C stood out strongly. The rows with high dislocation density were surrounded by a relatively low dislocation density, which made it difficult to assess an average defect density.

To determine whether a particular slip pattern dominated, the contrasts in the **g** = $1\bar{2}10$ and **g** = 00018 reflections were compared. We noticed that some contrasts visible in (c) were absent in the 00018 reflection (data not shown). To understand the variations in dislocation visibility, recall the visibility rules. Purely screw dislocations were invisible when the condition **g**·**b** = 0 was satisfied, where **b** is the Burgers vector. For the case of purely edge dislocations, the visibility was least when the conditions **g**·**b** = 0 and **g**·**n** = 0 were satisfied simultaneously, where **n** is a normal to the slip plane.

**Figure 2:**
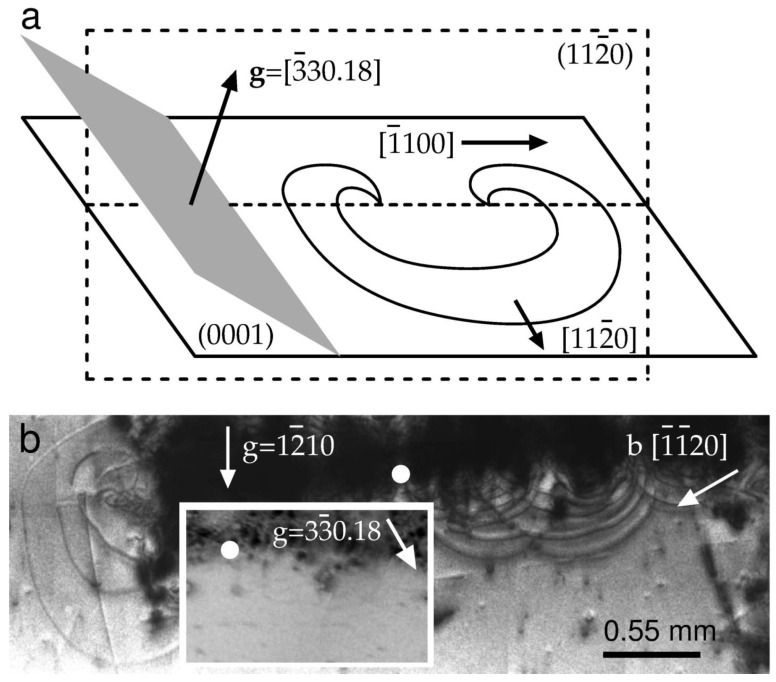
(**a**) Schematic relationship between the ($\bar{3}3018$) diffracting plane and dislocation semiloops emitted by the source on the basal plane. The Burgers vectors are parallel to 1/3 [$11\bar{2}0$]. (**b**) Magnified images of semiloops in the B region of Figure 1c. The inset shows the same region where the semiloop images are extinct in the $3\bar{3}018$ reflection. The solid circles denote the same spot on both of the topographs.

First, consider in detail those semiloops that expand away from the big pile labeled B in Figure 1c. The images of semiloops are shown enlarged in Figure 2b. The inset shows another reflection of the same area. The white circles indicate the same location on both of the topographs. The semiloops are visible in the **g_1_** = $1\bar{2}10$ reflection but invisible in the **g_2_** = $3\bar{3}018$ reflection; therefore, **g_2_**·**b** = 0, and the Burgers vector **b** of the semiloops is in the [$\bar{1}\bar{1}20$] direction. In addition, **g**·**n** = 0 because **b** lies on both the (0001) and ($3\bar{3}018$) planes.

A schematic representation of the semiloops along with the ($\bar{3}3018$) reflecting plane is shown in Figure 2a. The projection of **g** = $\bar{3}3018$ on the [$\bar{1}100$] direction shortens the length of **g** by a factor of cos 62° (i.e., by 0.5): **g**_project_ = 0.74 Å. A scalar product **g**·**b** equals 0, ∓3, and ±3 for the **b** vectors ±1/3 [$11\bar{2}0$], ±1/3 [$1\bar{2}10$], and ±1/3 [$\bar{2}110$], respectively. Since **g**·**b** = 0 and **g**·**n** = 0 are simultaneously satisfied in the $\bar{3}3018$ reflection, the dislocation semiloops in Figure 2a lie on the basal plane and belong to the (0001), [$11\bar{2}0$] slip system.

Second, we notice an imposing group of semiloops on the left of Figure 2b. The array contains segments of different visibility. However, when the $1\bar{2}10$, $3\bar{3}018$, or 00018 topographs are examined, the semiloops are contrasted for all three reflections. It is reasonable to assume that the array lies on the prism plane ($10\bar{1}0$) perpendicular to the growth direction. 

To get an idea of dislocation sources, let us recall that gas voids are nearly always present in sapphire ribbons. Voids are usually observed in thin surface layers located at a depth of about 100 μm [29]. In the present study, the back reflection geometry was employed to reveal dislocations in a thin slice of the ribbon close to the surface. In order to evaluate the observable depth *t*_obs._ of dislocations, we used the relationship between *t*_obs._ and the absorption depth *t_μ_* [30,31]. For low-absorbing crystals, the dislocation visibility in the back reflection was mainly determined by *t_μ_* with the assumption that the reflected intensity of the X-rays fell down to 10% of the incident power. Considering both paths of the incident and diffracted X-rays, *t_μ_* is calculated as follows [30]:

(1) $$t_{\mu }=\frac{\ln10\cdot \sin\omega }{\mu }\cdot \frac{1}{1+\sin\omega /\cos\left(90{}^{\circ}-2\theta +\omega \right)},$$

where *μ* = 18.147 cm^−1^, *ω* = 68° is the incident angle, and *θ* = 40.3° is the Bragg angle. The observable depth *t*_obs._ = 0.23 mm is about 1/5 of the ribbon thickness.

In the back reflection geometry, the strain fields of dislocations lying deeper in the crystal did not contribute significantly to the image. However, when the upper limit of the dislocation density of about 10^6^ cm^−2^ was reached, the overlap of the dislocation strain fields resulted in a darkening of the region near the dislocation sources. The intensity increase was due to the strong reflectivity of a defective crystal. Interestingly, as images of basal dislocations faded in the $3\bar{3}018$ reflection, some features became noticeable. Only features with a characteristic size of tens of microns were detected. We can suggest that when a void is large enough, it creates diffraction contrast caused by a change in thickness. As we will see later, oversized voids can merge into cavities.

Note that sources emitting dislocations on the basal plane of the sapphire were proved to operate as Frank–Read sources to emit basal dislocations [32]. Our topographic analyses revealed basal dislocations with the [$\bar{1}\bar{1}20$] Burgers vector direction. Dislocations with the ($10\bar{1}0$) prismatic slip plane were also observed. Those dislocations, which gave rise to pileups labeled A, B, and C, stood out strongly in all the obtained topographs. They might have had Burgers vector directions in the basal and prism planes. No experimental evidence of a predominant slip system in the specimens under consideration was found. Finally, in Figure 1 the [$10\bar{1}0$]—line-direction rows marked with arrows in (c) disappear in (d). These tangled dislocations, which are rendered invisible by the use of the back reflection, might lie deeper than the penetration depth of the X-rays.

### 3.2. Inhomogeneities

In the present work, micro-inhomogeneities in sapphire ribbons were revealed by the PCI method. Compared with optical microscopy, which is limited to thin slices due to the reduced focus length, the PCI technique provided high-quality images in thin and thick specimens. In the lensless scheme, a nearly parallel SR beam was used together with a pixelated detector. The spatial resolution of the image was related to the pixel size. When an optical lens magnified the image contrasts onto the CCD, the pixel-to-object size ratio was reduced. As a consequence, the FOV became smaller. An experimenter registered only a small fragment of the specimen shown by the detector. Eventually, the fragments could be assembled into a map up to several centimeters in size.

A topograph was recorded at a magnification of unity. The FOV size was shaped up due to slits, beam conditioners, and natural angular collimation of SR. The topographs were subsequently enlarged optically. In order to compare the topographs and the phase contrast images to find common features, a correct scaling needed to be determined. The scale showed a correlation between the topographic distances and the number of pixels in a digital phase contrast image. We emphasize that in the far-field conditions, the phase contrast image had a larger size compared with a small object. We further emphasize that far-field patterns were formed at rather small distances from a micro-object to the detector. Therefore, an inverse problem solution was necessary. Yet, it was always possible to measure large lengths and neglect the relative error.

**Figure 3:**
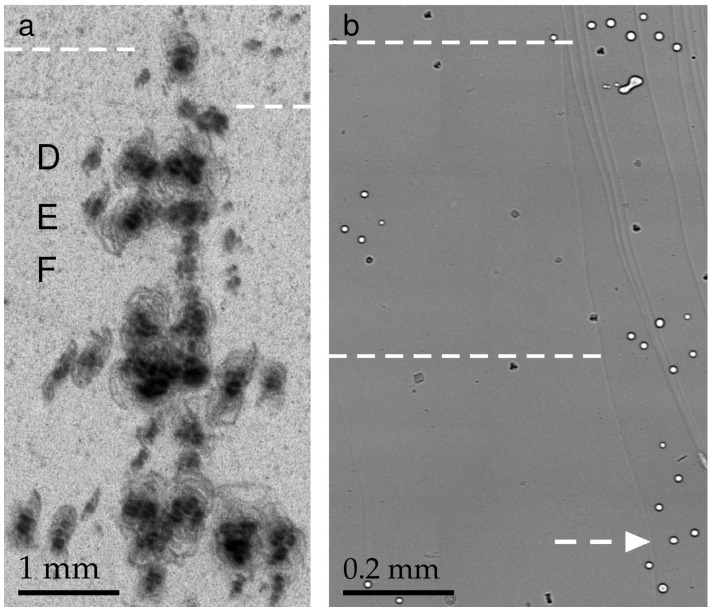
(**a**) General view of dislocations at the beginning of ribbon growth. The arrangement is revealed at the upper boundary of the box in Figure 1a. Lang topography; Ag*K*_α_ radiation, $\bar{3}300$ reflection of sapphire. (**b**) Phase contrast image of gas voids located in the same area with dislocations. The dashed lines cross each image at the same distance from the seed.

Figure 3 contains the same region of the ribbon imaged by XRT (a) and PCI (b) methods. The small spots that look darker than the background in (b) can be inclusions, generally formed during the growth process. Impurities in the melt, e.g., molybdenum (Mo) from the Mo shaper, are captured by the crystallization front [33]. We observed inclusions mainly in the neck portion. To our knowledge, the impurity effects on the generation of dislocations in the sapphire ribbons were not observed, and inclusions of foreign phases were not considered here. The gas void images in Figure 3b have a simple structure consisting of a black ring around the edge and a light color in the middle. Despite the fact that the distribution of large voids can be controlled by a number of growth parameters (see, e.g., [13,14,16]), a microvoid is still an extremely interesting object of research. There are very few works on the quantitative characterization of microvoids in sapphire crystals using X-ray imaging techniques. The present article fills this gap.

Several features are apparent in Figure 3: the tangles of dislocation semiloops labeled D, E, and F (a); the small voids of circular shape; and the groups of voids (b). In addition, a sparse distribution of inclusions between the voids and the as-grown surface relief is shown in (b). The dashed lines are located at the same distance from the seed. A direct comparison of the voids (b) and dislocations (a) shows that the voids were very much like the dislocations in their distribution and location. One can conclude that the voids were the cause of the dislocations.

Previous XRT experiments on sapphire ribbons were carried out in defective regions when the lattice distortion was high [16,17]. The nucleation centers were not visible because of an increased background intensity occurring when a crystal was imperfect. Unfortunately, the Bragg diffraction contrast was not adequate for studying the nature of defects, when the overlap of images prevented a meaningful interpretation. On the contrary, the PCI confirmed the assignment of the nucleation centers to gas voids. The relaxation of thermal stress began with the formation of glide dislocation semiloops at the voids. The same pattern was repeated further in the direction of growth.

**Figure 4:**
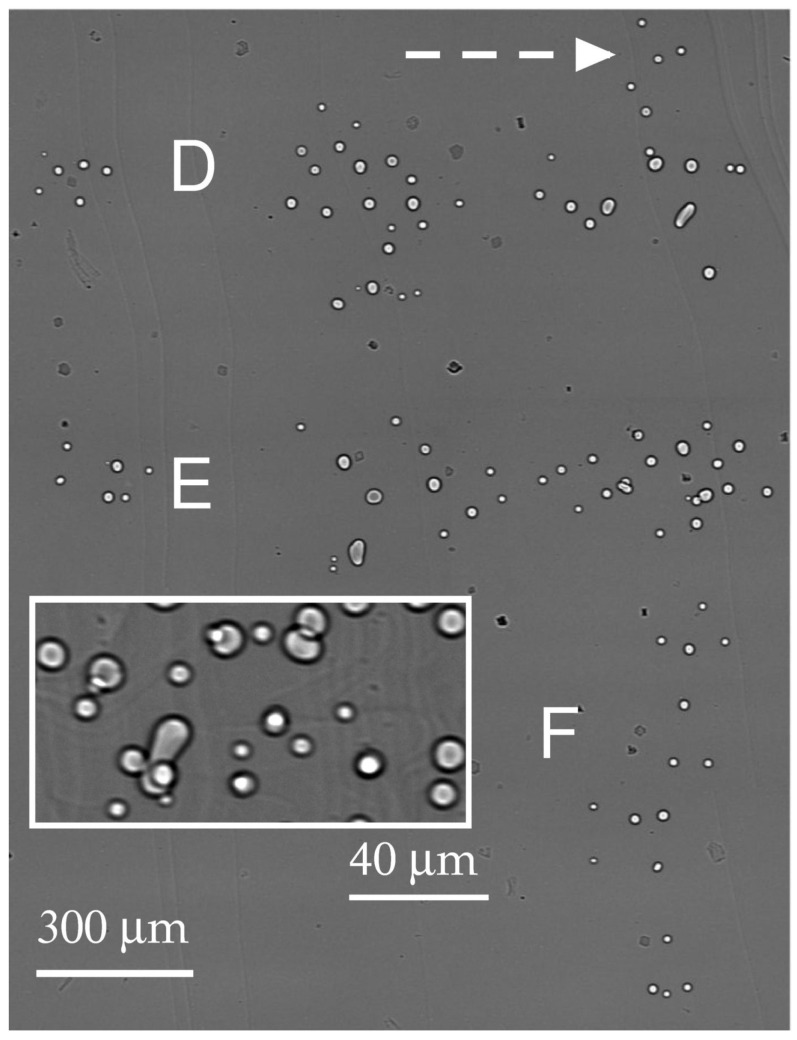
Gas voids in the part of the specimen later to grow as compared with the part in Figure 3b. The two parts match each other along the dashed arrow. The inset shows magnified images of voids farther away from the seed.

A small scale of the topograph in Figure 3a allows seeing almost the entire specimen area where defects were formed at the early stage of the ribbon growth. Compared with the topograph, the scale of the phase contrast images was larger. The same observable area is shown in Figure 3b and Figure 4. Each image was taken while looking down the growth axis from the seed. The images were connected according to the levels marked with dashed arrows. The distribution of gas voids was noteworthy: they were grouped and distributed across the width of the ribbon.

Consider first the sequences of the voids located between the left and the right of Figure 4. They are labeled D and E. The narrow gap between D and E is followed by a wider one. On the right, one sees some small voids (labeled F) running from the top to the bottom of the gap. A very similar motif can be found in the topograph below the dashed lines. D, E, and F denote rows of tangled dislocations (Figure 3a). Rows D and E, which lie across the ribbon width, are followed by row F running in the direction of growth. According to their size, the tangles were presumably generated by the larger (horizontal) and smaller (vertical) groups of voids.

Farther from the seed, the sequences of voids began to form in parallel to the direction of growth. The voids became closer, so that the SR beam, passing through the specimen, intersected pairs of voids. Such closely located configurations are shown in the inset in Figure 4. Under the action of thermal stress, voids can interact and merge. The coalescence can lead to the transformation of the shapes. Our observations indicated that the distortion of spherical shape proceeded gradually due to some changes in the growth conditions. The nature and evolution of non-spherical voids still remain unclear. In-depth understanding of this phenomenon requires the analysis of void sizes.

### 3.3. The Size of the Gas Voids

If an X-ray beam passed through a sample with inhomogeneous thickness and/or density, the wave field acquired an additional phase shift, variable in the direction perpendicular to the propagation direction. A void entailed a phase shift proportional to *s* Δδ, where *s* is the void thickness, and δ is a decrement of the refraction index *n* = 1 − δ of materials. In the hard X-ray range (*E* > 6 keV), the phase shift of a coherent SR beam could be detected when the sample thickness changed from at least ~0.1 μm to several microns. In the near field, i.e., at a short distance *z* from the sample, the intensity oscillations were simple in shape but varied in amplitude and period. For a larger *z*, the sinusoidal variation in the intensity specific to Fresnel zones was formed. The sizes of the microvoids could be determined using computer simulations. To make certain that the obtained cross section diameters are correct, one has to register and fit the images of the same void at some different distances *z* (see, e.g., [34,35]).

**Figure 5:**
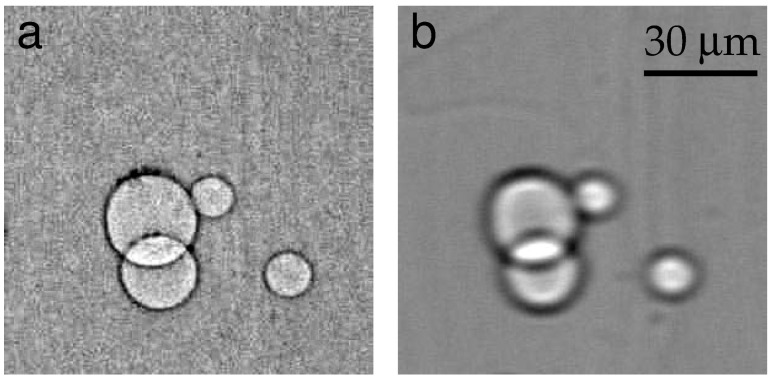
Experimental phase contrast images of some gas voids recoded at sample-to-detector distance *z*_1_ = 1.5 cm (**a**) and *z*_2_ = 20 cm (**b**).

As for spherical voids, their simulations were carried out in an earlier work [36]. Unlike these, we chose an experimental picture that showed the overlay of two voids located at a certain distance from each other. To our knowledge, the fitting of such configurations has not been reported. Figure 5 shows the phase contrast images recorded on the CCD at the distance *z*_1_ = 1.5 cm (a) and *z*_2_ = 20 cm (b). We note that the width of the void boundaries changed and became larger with the distance *z*, which was a consequence of the interference nature of the images. The boundaries did not overlap, so the void diameter could be roughly estimated from the image pixels. The preliminary estimates showed that the average diameter at both distances *z*_1_ and *z*_2_ was approximately 60 pixels. For a given pixel size of 0.325 μm, we obtained ≈20 μm. One can see that the diameters of the voids were greater than the diameter of the first Fresnel zone 2*r* = 2(*λ z*)^1/2^, which was equal to 2*r*_1_ = 2.2 μm and 2*r*_2_ = 8.1 μm at distances *z*_1_ and *z*_2_, respectively. The width of the edges increased with the distance *z*, but the average diameter did not change.

The inline phase contrast setup did not allow an evaluation of the depth of a void in a sapphire ribbon. It was, therefore, impossible to know the distance between voids since this distance did not influence the simulated images. The sizes of voids can be different. Below we will consider a model applicable to a specific case, namely, two identical spheres with a diameter of 20 μm. Their centers were shifted diagonally by 8 × 2^1/2^ = 11.3 μm. The calculation was performed by the XRWP (X-ray Wave Propagation) program [37]. The program calculated a series of two-dimensional patterns using formulas of the phase contrast theory of three-dimensional objects. The wave propagation through a substance, in which the electron density varied, was described by the transmission function of the object. Since the object containing voids was not uniform, the thickness was a variable function of the coordinate. Propagation in a free space was calculated according to the Huygens–Fresnel principle as the convolution of the wave function with the Fresnel propagator [1]. The convolution was computed through the Fourier transform method. The fast Fourier transformation (FFT) method was applied [38].

Prior to describing the results of the simulation, it is necessary to emphasize that for the imaging techniques, the recorded image resulted from a convolution of the sample response and the detector characteristics. In addition, the experimental pattern depended on the size of the SR source. A good approximation of the source is the model in which every point of its transverse size radiates independently, and the radiation intensity obeys the Gaussian law, i.e., the source may be characterized by the half-width of the Gaussian function. The structure of an image also depended on statistical and instrumental noise. The signals recorded using a CCD were superimposed on a background. When the calculated pattern was compared with the experimental one, it was necessary to normalize the latter. In order to obtain a good fit between the simulation and the experiment, the constant background must be subtracted.

**Figure 6:**
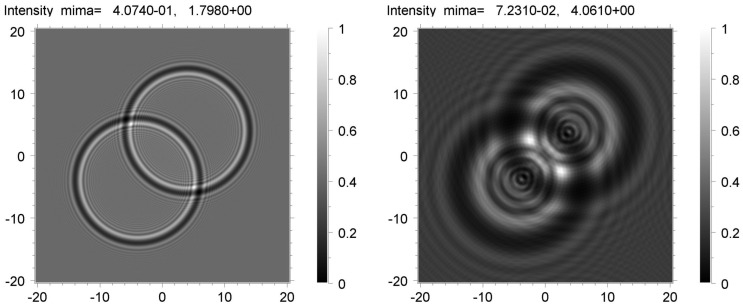
Theoretical phase contrast images of two spheres with 20 μm diameter calculated for the distances *z*_1_ = 1.5 cm (**left**) and *z*_2_ = 20 cm (**right**).

At the initial stage, the simulation was carried out for fully coherent monochromatic radiation emitted by a point SR source and recorded using an ideal detector. The program calculated theoretical images, which contained sharp peaks of high intensity. The sample area containing these peaks was very small. A comparison of the image on the left in Figure 6 with the image on the right shows that the effect became more pronounced at the long distance *z*_2_ = 20 cm. Prior to making comparisons with experimental images, the calculated images were averaged. We employed the convolution of the two-dimensional intensity distribution on the detector with a two-dimensional Gaussian function. Since the asymmetry of the directions along and across the SR source was not noticeable, we used the symmetric two-dimensional Gaussian. The convolution was performed for different values of the full width at half maximum (FWHM), which were the same in two directions.

**Figure 7:**
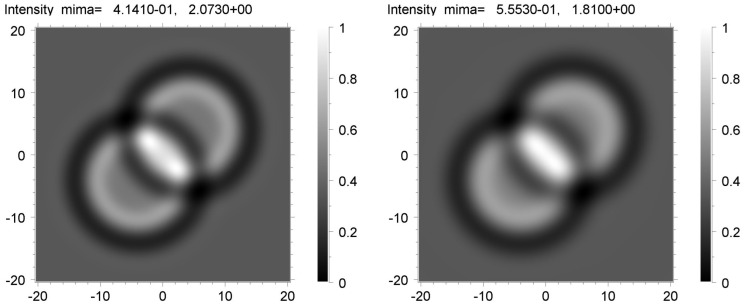
Phase contrast images calculated for the distance *z*_2_ = 20 cm and averaged using convolution with Gaussian of FWHM = 3 μm (**left**) and 4 μm (**right**).

The best fits to the experimental data were selected. Figure 7 shows the averaged theoretical images of the voids calculated for the distance *z*_2_ = 20 cm using the convolution with the Gaussian of the FWHM = 3 μm (left) and 4 μm (right). Let an optimal Gaussian FWHM be 3.5 μm. We emphasize that the value of FWHM must depend on the distance *z*. There were, therefore, good reasons for the automatic variation in two parameters during the simulation: the value of the FWHM and the sample-to-detector distance *z*. If we assume that the averaging occurred due to the source size, a decrease in *z* by a factor of 13.3 caused a decrease in FWHM by the same factor. Thus, we might expect that for the distance of 1.5 cm, an optimal FWHM was 0.26 μm.

**Figure 8:**
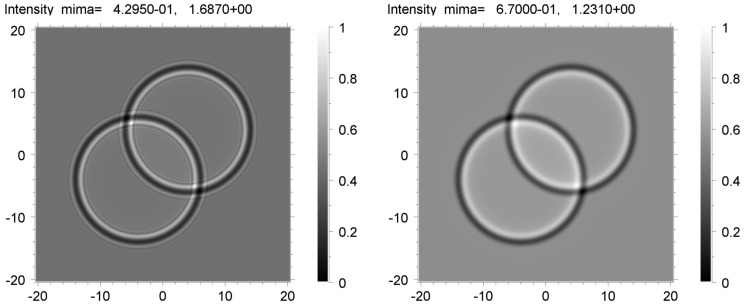
Phase contrast images calculated for the distance *z*_1_ = 1.5 cm and averaged using the Gaussian of FWHM = 0.26 μm (**left**) and 1 μm (**right**).

However, this effect is not observed in Figure 8, which contains the image simulations by FWHM = 0.26 μm (left) and 1 μm (right). The theoretical image on the right exhibits the distinctive effect of a large FWHM. It is more consistent with the experimental picture than the image on the left in Figure 8. To sum up, the averaging arising from the source size was observed but only to a certain extent. The given example clearly shows that imaging detectors and other aspects of the experimental procedure (including a sample vibration) should not be neglected when extracting quantitative information from an image.

## 4. Discussion

The generation of dislocations on voids in sapphire ribbons is obviously related to thermal stress relaxation at the stage of crystal cooling. For the basal slip of dislocations in the sapphire, there is an empirical formula that determines the critical shear stress *τ_cb_* in the dependence of the temperature [27]:

(2) $$\ln\tau_{cb}=\ln\tau_{0b}-0.0052T,$$

where *τ*_0*b*_ = 109 GPa and *T* is the absolute temperature given in Kelvins. For example, at *T* = 2273 K, (2) results in *τ_cb_* ≈ 0.8, which is of the order of magnitude of the thermal stresses in the growing sapphire crystals in the pre-melting state [16]. Thus, the dislocations can easy glide in this state of the growing sapphire crystals. However, the process of their nucleation needs careful examination.

Lubarda et al. [39] considered the emission of a straight edge dislocation from a cylindrical void under uniform biaxial tensile stress *σ* and showed that the critical stress value required to emit the dislocation was

(3) $$\frac{\sigma_{cr}}{G}\geq \frac{b/R}{\sqrt{2}\pi \left(1-\nu \right)}\frac{{\left(1+\sqrt{2}\rho b/R\right)}^{4}+1}{{\left(1+\sqrt{2}\rho b/R\right)}^{4}-1},$$

where *G* is the shear modulus, *b* is the Burgers vector magnitude, *R* is the void radius, *ρ* is the normalized cutoff radius, *ρ* = *w*/*b*, for the dislocation stress field at the dislocation core, and *w* is the width of the core. It is seen that the critical stress *σ_cr_* decreases with an increase in the void radius *R*. However, when *R* >> *b* (this is our case), this dependence saturated, and *σ_cr_*/*G* → 1/[4*π* (1 − *ν*)*ρ*]. For typical values *ν* = 0.21 and *ρ* = 1, this resulted in *σ_cr_* ≈ *G*/10. This characteristic value was extremely high and not available under the crystal growth conditions.

Yan et al. [40] suggested an alternative approach for estimating the critical stress needed for dislocation emission from a void. They noticed that in reality, the voids were faceted, and therefore, one can expect the dislocation emission from the edges at the void surface. In this case, “the critical length determining dislocation nucleation is not the void diameter but the length of the void edge and the stress to nucleate the dislocation scales with this length, similar to that from a Frank–Read source” [40]. As a result, the authors came to the following formula for the critical shear stress:

(4) $$\tau_{cr}=\frac{Gb}{L},$$

which is similar to that describing the activation of a Frank–Read source. Here *L* is the length of the void edge. Since the largest edge of a faceted void is roughly equal to the void radius, *L* ≈ *R*, for *R* = 10 μm and *b* = 0.5 nm, (4) results in *τ_cr_* = 5 × 10^−5^ *G*.

At relatively low temperatures, the shear modulus of sapphire is about *G* ≈ 150 GPa, which would provide us the value of *τ_cr_* ≈ 7.5 MPa. This value is approximately 10 times higher than the magnitude of the thermal stresses in growing sapphire crystals in the pre-melting state [16]. However, in this state, the shear modulus also can be smaller by the order of magnitude than that under low temperatures [41].

Thus, one can conclude that dislocations can be generated by the edges of faceted voids under the action of the thermal stresses in growing sapphire crystals. The inhomogeneous distribution of these stresses across the growing crystals [16] is responsible for the inhomogeneous generation of dislocations by the voids, when the voids in some regions of the crystal do emit the dislocations, while the voids in other neighboring regions do not. The role of internal gas pressure in the voids on the process of dislocation emission needs special consideration.

## 5. Summary

High technical standards for basal-faceted sapphire ribbons intended for windows, mirrors, and substrates make it necessary to improve single crystallinity and perfection of these products. XRT and PCI is a powerful combination to understand the relationships between the defects and inhomogeneities in crystals. The topographs and phase contrast images obtained with monochromatic SR beam allowed us to directly relate dislocations with gas voids.

By using large-area mapping of voids, we found that once nucleated, they tended to group and to spread across the width of the ribbon. When the amount of voids in the growing ribbon increased, they spread along the growth direction as well. The resulting distribution pattern was similar to a “grid” structure. Single spherical microvoids did not possess a dislocation generating activity. Moreover, the determination of void sizes using computer simulations revealed a low activity of larger spherical voids as well. In contrast, dislocations were associated with the strain fields of nonspherical cavities. The analysis of the data suggested that the voids united to form larger aggregates, which eventually developed into cavities of heterogeneous shape and slightly irregular surface. Particularly, the XRT data showed that the dislocation density increased and that the density of the dislocation sources became much higher in association with the “grid” pattern, especially in the vicinity of the dense groups of voids. At the initial stage of plastic deformation, no other mechanisms were observed.

Dislocation semiloops located near the centers of their generation were shown by XRT. A topographic **g**·**b** analysis revealed basal dislocations with 〈$1\bar{2}10$〉 Burgers vectors. Prismatic semiloops belonging to the ($10\bar{1}0$), [$1\bar{2}10$] slip system were also present. The curved dislocation lines tended to assemble into tangles, in which no unambiguous determination of the slip system was reached because of the high density of dislocations. In addition, peculiar changes in the visibility of some tangled dislocations suggested that they were located at different depths. No evidence of the predominant slip system in the specimens under consideration was found.

## Data Availability

Data are contained within the article.
